# The applications of CRISPR/Cas system in molecular detection

**DOI:** 10.1111/jcmm.13925

**Published:** 2018-10-19

**Authors:** Li Zhou, Rongxue Peng, Rui Zhang, Jinming Li

**Affiliations:** ^1^ National Center for Clinical Laboratories Beijing Hospital National Center of Gerontology Beijing China; ^2^ Graduate School Peking Union Medical College Chinese Academy of Medical Sciences Beijing China; ^3^ Beijing Engineering Research Center of Laboratory Medicine Beijing Hospital Beijing China

**Keywords:** CRISPR/Cas systems, diagnostics, molecular detection, pathogens, single nucleotide variants

## Abstract

The *Streptococcus pyogenes* CRISPR/Cas system has found widespread applications as a gene‐editing and regulatory tool for the simultaneous delivery of the Cas9 protein and guide RNAs into the cell, thus making the recognition of specific DNA sequences possible. The recent study that shows that Cas9 can also bind to and cleave RNA in an RNA‐programmable manner is suggestive of potential utility of this system as a universal nucleic‐acid recognition tool. To increase the signal intensity of the CRISPR/Cas system, a signal amplification technique has to be exploited appropriately; this requirement is also a challenge for the detection of DNA or RNA. Furthermore, the CRISPR/Cas system may be used to detect point mutations or single‐nucleotide variants because of the specificity of the recognition between the target sequence and the CRISPR/Cas system. These lines of evidence make this technique capable of detecting pathogens during infection via analysis of their DNA or RNA. Thus, here we summarize applications of the CRISPR/Cas system to the recognition and detection of DNA and RNA molecules as well as the signal amplification. We also describe its potential ability to detect mutations and single‐nucleotide variants. Finally, we sum up its applications to testing for pathogens and potential barriers for its implementation.

## 1 INTRODUCTION

Clustered regions of interspersed palindromic repeats with Cas9 (CRISPR/Cas9) is an antiviral defense system first found in *Escherichia coli* in the 1980s.^1^ It started to emerge as a powerful technique for gene editing after clarification of the mechanism of the type II CRISPR system that needs only one Cas protein to cleave target sites.^2^ Jinek et al chose the CRISPR system of *Streptococcus pyogenes*, which involves a single Cas protein (Cas9) and two RNAs (crRNA [CRISPR RNA] and transactivating crRNA, also known as tracrRNA) to build an active CRISPR/Cas endonuclease complex. The researchers found it possible to combine these two RNAs into a chimeric single guide RNA (known as sgRNA) that can effectively direct Cas9 to specific DNA targets. The rules used by Cas9 to search for a DNA target are not complicated, the specificity is determined only by a 20‐nucleotide (nt) sequence in the sgRNA that hybridizes with the target DNA in the presence of a DNA protospacer‐adjacent motif (PAM) in the complementary region close to the target site.^2^, ^3^ By binding to the DNA target sites, the sgRNA‐Cas9 complex can create a double‐strand break which can be repaired by non‐homologous end joining or by the homology‐directed repair pathway.^4^, ^5^, ^6^ The first pathway usually introduces insertions and/or deletions (indels), which will alter open reading frames and insert premature stop codons resulting in a gene knockout. After a double‐strand break, prevalence of the action of the homology‐directed repair pathway, which needs the presence of a donor DNA template that ensures precise gene correction or recombination, is very low in comparison with the non‐homologous end joining pathway. The 20 nucleotides in the sgRNA are customizable,^2^ which makes the design of sgRNA easy in practice, greatly expanding its applications in biological research. In this review, we provide a brief summary of the applications of the CRISPR/Cas system to molecular detection including detection of specific sequences of DNA or RNA, single nucleotide variants and pathogens.

## 2 APPLICATIONS TO DETECTING SPECIFIC SEQUENCES OF DNA OR RNA

**Figure 1:**
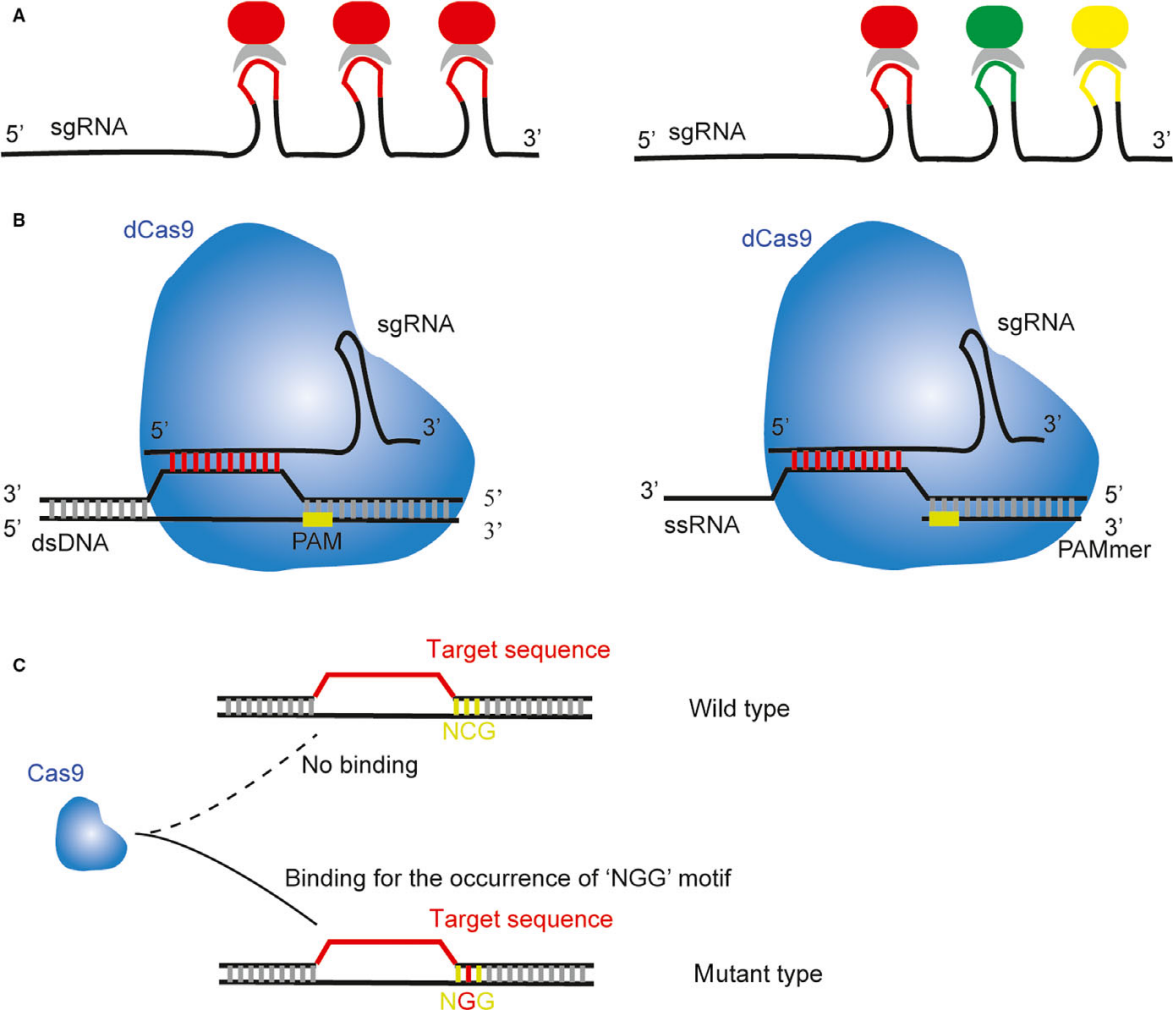
The applications of CRISPR/Cas system in molecular detection. A, Extension of the 3′ end of sgRNA to add extra hairpins. The RNA‐binding proteins (RBPs) are grey. The fluorescent proteins (FPs) are highlighted in red, green, and yellow. The left‐hand panel shows signal amplification effects of the addition of an extra hairpin structure for increasing the number of FPs for labelling the RBP. The right‐hand panel illustrates application of multicolour labels via different hairpin structures and a corresponding RBP and FP. B, Customization of dCas9/sgRNA for binding to DNA or RNA. The left‐hand panel shows that recognition of the double‐stranded DNA (dsDNA) by the sgRNA is determined by the 20 specific nucleotides of the sgRNA and by the PAM motif on the complementary strand close to the target region. The right‐hand panel shows that the artificial PAMmer composed of a deoxyribonucleotide is designed to assist sgRNA in recognition of a single‐stranded RNA (ssRNA), because there is no PAM motif in the ssRNA. C, One of the results of a single‐nucleotide variant (SNV) is creation of a novel PAM because of the appearance of the “NGG” motif, which makes the binding between Cas9 and the mutant DNA possible

DNA fluorescence in situ hybridization (FISH) has been widely used to visualize sequence‐specific genes for research and diagnostic purposes. Nonetheless, it requires heat treatments and formamide to denature double‐stranded DNA (dsDNA) to enable probe hybridization; these treatments may affect integrity of the biological structure and organization of the genome. Accordingly, Baohui Chen^7^ employed an enhanced green fluorescent protein (EGFP)‐tagged dCas9 protein and a structurally optimized sgRNA to image repetitive elements and non‐repetitive genomic sequences. They modified the sgRNA design to enhance its stability and to promote its assembly with the dCas9 protein. They substituted an A‐U base pair flip with a putative Pol‐III terminator in the sgRNA stem loop to avoid premature termination of U6 Pol‐III transcription and extended the dCas9‐binding hairpin structure to improve sgRNA‐dCas9 binding. Both modifications increased the targeting efficacy and decreased background and nucleolar signals. They combined the two modifications and found that this approach not only improves the efficacy of imaging but also increases the efficiency of gene regulation. They designed 73 sgRNAs targeting both DNA strands spanning a 5 kb non‐repetitive region of the *MUC4* gene and concluded that 26 to 36 sgRNAs are sufficient to detect a non‐repetitive genomic locus by means of CRISPR. They also labelled different regions of the same gene or different genes with multiple sgRNAs and reported that the spots of imaging via two genes increased whereas those of the same gene did not, suggesting that CRISPR can label multiple genomic elements at the same time. On the basis of this observation, Wulan Deng^8^ think up a way to directly detect a sequence of interest in a genome without denaturation of dsDNA by applying the dCas9/sgRNA complex to FISH in fixed cells on account of this complex's strong and stable affinity for its target DNA. They coexpressed a fluorescent protein with the dCas9 protein and utilized the direction of sgRNA with a target sequence to detect and visualize specific sequences of DNA. Both the major satellite and telomere that contain hundreds to thousands of repeats and some repetitive sequences with tens to hundreds of copies for sgRNA targeting were imaged successfully in fixed cells. Nevertheless, as to the non‐repetitive sequences, the labelling efficacy of the target gene was low and the background signal was quite strong, suggesting that sgRNA's targeting efficacy should be optimized for a non‐repetitive sequence. This is because the fluorescent signal of a few dCas9‐sgRNA complexes in the target region was insufficient for detection. According to the research of Baohui Chen,^7^ at least 26 unique sgRNAs are needed to target the same region to obtain a detectable signal. On the other hand, this approach has been difficult to practice in biological applications because of the challenges in the delivery of dozens of sgRNAs into the cell and large numbers of off‐target sites associated with the great many sgRNAs. Thus, Peiwu Qin^9^ devised a robust fluorescent‐signal amplification system by utilizing a re‐engineered sgRNA, which contains up to 16 MS2 motifs that can bind to bacteriophage MS2 coat protein (MCP), so that they can label the target via a fluorescent tag of MCP (Figure 1A). The researchers improved the traditional method of targeting non‐repetitive regions by employing four unique sgRNAs rather than 26 to 36 sgRNAs because the amplification effects of the large number of fluorescently tagged MCP molecules can reduce the number of dCas9/sgRNA complexes required for reliable detection. They also determined that a further reduction in the number of sgRNAs may be possible, whereas the potential loss of dCas9 targeting specificity owing to the modification of sgRNA should be taken into account. Apart from the signal amplification and single‐colour labelling technique, multicolour CRISPR labelling of chromosomal loci has been developed: Hanhui Ma et al^10^ used three orthogonal CRISPR/dCas9 components tagged with different fluorescent proteins to detect multiple loci in the genome so that this method can differentiate various chromosomal loci simultaneously and confirm the distance between the loci at the same time (Figure 1A). The limitation of this approach is that apart from *S. pyogenes* Cas9 whose PAM is required only for the “NGG” sequence, many other bacterial Cas9 proteins recognize more complicated PAM sequences, which makes the target sequence more demanding. Consequently, to circumvent these issues, Yi Fu et al^11^ developed a new method for labelling a target site not by fluorescently tagged dCas9 but by a newly engineered sgRNA containing RNA stem loop motifs MS2 and PP7 that can bind to bacteriophage coat proteins MCP (MS2 coat protein) and PCP (PP7 coat protein). Furthermore, the RNA‐binding viral proteins MCP and PCP can be labelled with different colours via fluorescent proteins so that two distinct targets can be visualized simultaneously. As in Yi Fu's study, Siyuan Wang^12^ conducted analogous research and found that extending the tetraloop and stem loop 2 of the sgRNA with MS2 or PP7 aptamers can increase the signal‐to‐background ratio of chromatin imaging. In the meantime, Hanhui Ma et al^13^ proposed an approach similar to but more advanced than the above method. They selected two of three hairpins (MS2, PP7, and boxB) to fuse to stem loops or to the 3′ end of the sgRNA, and this approach yielded six combinations. Therefore, the final sgRNAs were capable of recruiting six different pairs of fluorescent proteins fused to RNA hairpin‐binding proteins, which can recognize the corresponding RNA elements. In the end, six colours were presented through different combinations. Thus, the simultaneous imaging of six chromosomal loci was feasible with this approach.

In view of the development of DNA detection technologies, from repetitive to non‐repetitive sequences, the challenge was to enhance the signal released from the locus of the target sequence labelled with re‐engineered sgRNA/dCas9–viral RNA‐binding protein–fluorescent protein complex. Comparison of the detection of repetitive and non‐repetitive sequences revealed that the former yields an obvious signal amplification effect because its abundant copies can combine with much more sgRNA molecules, which should produce a stronger signal. Nonetheless, the latter was not able to produce such a strong signal this way. Therefore, the researchers devised two other methods to amplify the signal: one involves transduction of large amounts of sgRNAs targeting the same gene in different regions, and the other involves attaching more fluorescent proteins to one single sgRNA. The limitations of the first method are the following: (a) with the increase in the number of sgRNAs, the off‐target effects will become more serious and ineluctable; and (b) it is technically difficult to deliver so many sgRNAs into a cell. It seemed that the second method is suitable for detection of a single non‐repetitive sequence but still required four sgRNAs, and lattice light sheet microscopy was needed to obtain a reliable signal. From the single loci to simultaneous multilocus detection, various improvements have been made to add various fluorescent colours though the method was still restricted to six colours. Accordingly, there is still much room for improvement.

One study indicates that the HNH nuclease domain of Cas9 is homologous to other HNH domains that cleave RNA substrates.^14^ Doudna^15^ lab combined this result with their prior discovery^16^ that single‐stranded DNA (ssDNA) targets can be activated for cleavage by a separate PAMmer (an oligonucleotide that hybridizes to the target and function as the PAM). They wondered whether Cas9 can cleave ssRNA targets while the PAMmer is present on the complementary side. Thus, they tested a series of DNA and RNA substrates in vitro cleavage experiments and made a conclusion that deoxyribonucleotide‐containing PAMmers can specifically activate Cas9 to cleave ssRNA, whereas ribonucleotide‐based PAMmers cannot. They also found that even without the PAMmer, dCas9 can bind to the target ssRNA but the binding affinity is much weaker than with a PAMmer, and the high‐affinity binding may not need correct base pairing between the guide RNA and the ssRNA target, especially if the complementary PAMmer is present. What's more, the extension of the 5′ end of a PAMmer can improve the binding specificity between the guide sequence and the PAMmer‐ssRNA target while binding affinity and cleavage efficacy may undergo concomitant losses. Most importantly, their lab demonstrated that Cas9 can be specifically directed to bind or cut RNA targets while avoiding corresponding DNA sequences via custom design of the PAMmer sequence. In addition, they tried to apply this approach to isolate endogenous *GAPDH* transcripts from a cell lysate under physiological conditions. At first, they obtained only two *GAPDH*‐specific RNA fragments. But after complete elimination of RNase H–mediated RNA cleavage, they successfully isolated intact *GAPDH* mRNA and observed that in the absence of a PAMmer, *GAPDH* mRNA can still be isolated though with lower efficacy. These data indicate that the Cas9/gRNA complex binds to *GAPDH* mRNA through direct RNA–RNA hybridization. These results mean that this approach can help to purify endogenous untagged RNA transcripts.

The study on CRISPR/Cas9 targeting of RNA began to gain popularity after Doudna's study. David M. Shechner^17^ developed a method for locus‐specific targeting of long non‐coding RNAs in vivo, and this approach is called CRISPR‐Display. This method uses a nuclease‐deficient *S. pyogenes* Cas9 mutant (dCas9) to deploy a large RNA cargo to targeted DNA loci by incorporating the cargo into the sgRNA. In other words, the sgRNA became a longer sgRNA that contains a sequence of interest such as artificial aptamers, pools of random sequences, and natural long non‐coding RNAs. The sgRNA may bind to genomic loci so that finally we could find out where the RNA sequence of interest is localized. The limitation of this method is that the length of inserted RNA should be at least 4.8 kb. This approach is different from the procedure for inserting the RNA sequence into sgRNA to display RNA domains. The Doudna lab quickly demonstrated that nuclease‐inactive CRISPR/Cas9 can bind RNA in a nucleic‐acid–programmed manner and allows for endogenous RNA tracking in live cells^18^; this approach was more convenient than the design of new sgRNA. The researchers called this nucleus‐localized RNA‐targeting Cas9 “RCas9” and confirmed that RCas9 can track RNA to oxidative‐stress–induced aggregates of RNA and/or proteins that are thought to be involved in neurodegeneration.^19^ These findings remind us that RNA tracking and targeting may give us quite a convenient way to explore the basic pathogenesis and pathological processes of diseases. Because all cells of an individual contain almost the same DNA, the functional distinctions between cell types are closely related to the portions of the genome that are transcriptionally active. As a result, expression of RNA is linked to many diseases. For example, the expression of certain small non‐coding RNAs known as microRNAs is increasingly recognized as a characteristic feature of oncogenic transformation. Tumour microRNA signatures can act as biomarkers that show the type of cancer and associated clinical outcomes.^20^, ^21^

## 3 APPLICATIONS TO THE DETECTION OF MUTATIONS AND SINGLE‐NUCLEOTIDE VARIANTS (SNVs)

It is well known that CRISPR/Cas9 has high efficacy of site‐specific gene targeting, but its potential off‐target effects have raised major concerns regarding its application in many respects. What's more, in Luhan Yang et al^22^ experiments, they first observed that a common SNV in the human genome may create a frequent Cas9 off‐target site. They demonstrated that a single germline SNV can create a recurrent off‐target site that might be missed in in silico predictions based on a reference genome sequence. This situation made the detection of a point mutation or single‐nucleotide polymorphism (SNP) a serious challenge. The cleavage site specificity of the sgRNA/Cas9 complex is determined by a DNA sequence that is complementary to the sgRNA and is flanked by a short PAM. Hence, if the SNV only happened to the target DNA sequence that is complementary to the sgRNA or the PAM sequence, two types of outcome may ensue: introduction of a mismatch between the guide RNA and the target DNA or disruption of the target PAM sequence.^23^ Both consequences may result in off‐target effects. Nevertheless, this phenomenon also provided us with two ideas to examine the already happened point mutations or SNP as we mention below. The first method is based on the match between the sgRNA and the mutated target DNA (Figure 1B), the second derives from the formation of a novel PAM (Figure 1C).

Techniques have been developed to detect mutations, for example, Surveyor nuclease assays and T7 endonuclease 1 (T7E1) assays. Both are enzyme mismatch cleavage assays for detection of a single‐base mismatch or small indels and target and cleave mismatched heteroduplex dsDNA. Then, the digested DNA fragments can be distinguished by agarose gel electrophoresis to determine whether there is a mutation. Nonetheless, the limitation of the two assays is that they cannot differentiate homozygous biallelic mutant clones from wild‐type clones and heterozygous monoallelic mutant clones from heterozygous biallelic mutant clones. Jong Min Kim et al^24^ reported a genotyping method that can increase the resolution and solve the above problem. They employed CRISPR/Cas‐derived RNA‐guided engineered nucleases (RGENs) in restriction fragment length polymorphism (RFLP) analysis for cleaving the target sites to get rid of limitations on restriction sites in RFLP analysis because of the specificity and editability of the sgRNA/Cas9 complex. It was demonstrated that the newly discovered RGEN‐RFLP analysis can be applied not only to genotyping of indels induced by the CRISPR/Cas9 system but also those caused by some other gene‐editing tools. The authors also utilized a human colorectal cancer cell line (HCT116) that carries a gain‐of‐function 3 bp deletion to test whether this assay can analyse naturally occurring variations. The results showed that PCR products amplified from the cells harbouring only wild‐type alleles were cleaved by the wild‐type–specific RGEN and were not cleaved at all by the mutation‐specific RGEN. Meanwhile the heterozygous were cleaved partially by both wild‐type‐specific and mutant‐specific RGENs; this approach reveals the possibility of analysing mutations. In contrast, the results on the HEK293 cells harbouring a 32 bp deletion (del32) indicated that the del32‐specific RGEN cleaves the PCR products from wild‐type cells as effectively as those from HEK293 cells, reminding us about the inaccuracy of this cleavage. Lately, they found that this RGEN has an off‐target site with a single‐base mismatch downstream of the on‐target site, which means that this method is not specific enough to distinguish sequences with SNVs owing to their off‐target effects. This author decreased RGEN activity by means of a single‐base–mismatched guide RNA instead of a perfectly matched RNA such that the RGEN can discriminate between a wild‐type sequence and mutant sequence via a single base. Incidentally, they have shown in another paper that it was two single bases not one base that the RGEN needs to discriminate on‐target sites from off‐target sites.^25^ They created one more base mismatch deliberately to distinguish the wild‐type sequence from a mutant sequence and succeeded. They have confirmed that the new method can genotype three recurrent oncogenic point mutations in genes *KRAS*,* PIK3CA* and *IDH1* in human cancer cell lines by means of the activity‐decreased RGENs. Moreover, they can also detect point mutations in the *BRAF* and *NRAS* genes using RGENs that recognize the “NAG” PAM sequence.

It is not easy to control the recognition and cleavage of the RNA‐guided Cas9 nuclease because its specificity is dependent on the length of the sgRNA^26^ and on the sequence, number, position and distribution of mismatches.^27^ Courtney^28^ published a new procedure for targeting a point mutation or SNP because the Cas9 protein has quite a specific DNA‐binding constraint, namely, the target sequence in genomic DNA must be followed by the correct PAM sequence: 5′‐NGG‐3′. In addition, they found a mutation within *KRT12* that causes Meesmann's epithelial corneal dystrophy and can lead to the occurrence of a novel PAM. This meant that they could design a sgRNA complementary to the sequence adjacent to this mutation‐derived PAM to target and cleave the mutated gene. They took advantage of this approach in treating Meesmann's epithelial corneal dystrophy. Another team^29^ also applied this method to the human *KRAS* gene to solve the problem of acquired drug resistance to a MEK signalling inhibitor. Both confirmed that the mutation‐ or SNP‐derived PAM could be put to good use in the treatment of some dominantly inherited diseases because the sgRNA/Cas9 complex can specifically disrupt the mutant allele, whereas expression of the wild‐type allele would remain unaffected.

Let us review the two assays that are aimed at recognizing an SNV that happened in a genome. An SNV generally has two kinds of consequences: one is the appearance of a new target gene that is different from the former target gene by a single base (relevant to the high specificity of the modified sgRNA/Cas9 complex). The other is the emergence of a novel PAM of Cas9 if there is an ‘‘NGG’’ sequence resulting from the SNV. As for the new target gene, researchers find that by introduction of a single‐base mismatch into the guide RNA, the cleavage specificity of Cas9 can be improved probably because of the decreased enzymatic activity of RGEN, which is quite interesting and needs to be clarified. This assay could be used to distinguish between a mutant type and wild‐type, and thus, three recurrent oncogenic point mutations in genes *KRAS*,* PIK3CA* and *IDH1* in human cancer cell lines have been identified. As for the novel PAM, the cleavage activity of Cas9 is often employed to disrupt the mutated gene while the wild‐type allele is unaffected. This method is more likely to cure some heterozygous mutations causing an autosomal dominant (AD) single‐allele disease because of the subsequent homology‐directed repair after cleavage by Cas9.

## 4 DETECTION OF PATHOGENS

Emerging infectious diseases, such as Zika virus (ZIKV) infections in Latin America and Southeast Asia, have rapidly drawn attention to the need to develop simplified diagnostic tests for clinical practice in some underdeveloped geographic areas. Some authors have published papers about applying the CRISPR/Cas system to the molecular diagnosis to detect the presence of virus such as Zika virus. Keith Pardee^30^ reported a novel technique based on the sequence‐specific nuclease activity of CRISPR/Cas9 to distinguish between ZIKV strains with single‐base discrimination, for which the presence of an “NGG” PAM is necessary for the specific cleavage by Cas9. There are many strain‐specific PAM sites among Zika virus strains, such as African and American Zika variants. The authors exploited the (ds)DNA, an intermediate of the NASBA (Nuclear Acid Sequence Based Amplification) amplification process, as a substrate for the Cas9 endonuclease, leading to the production of either truncated or full‐length trigger RNA, which can differentially activate a toehold switch. If Cas9 cuts this dsDNA intermediate, and the target RNA is not amplified intactly so that it cannot trigger the toehold switch, then this situation reveals that the strain has the specific site in question. The discrimination of different Zika variants is useful for genotyping and determining the origin of an infection. Besides, infections by a variant Zika virus may have different clinical manifestations.^31^ Nonetheless, sometimes it is necessary for diagnostic platforms to accept some genetic mutations because of an evolutionary drift. That study also confirms that their assay can tolerate the expected genetic variation found in nature. Those variant strains have the ability to fully activate toehold sensors with up to 4‐nt (11%) mismatches.

In contrast, Deng Wulan et al^8^ combined the nuclease‐deficient Cas9 (dCas9) with various sgRNAs to localize the pericentromeres, centromeres, and telomeres without denaturation of dsDNA so that the integrity of the biological structure and organization of the genome was not disrupted. Kyeonghye Guk^32^ found a way to detect methicillin‐resistant *Staphylococcus aureus* (MRSA) using a CRISPR‐associated protein 9/single‐guide RNA (dCas9/sgRNA) complex as a targeting material to capture genomic DNA of MRSA by specific recognition of the *mecA* gene (which is implicated in the resistance to methicillin in MRSA^33^) by the sgRNA. Because genomic DNA of most methicillin‐susceptible *S. aureus* (MSSA) strains does not contain the *mecA* gene, the well‐designed dCas9/sgRNA complex should not affect MSSA. Next, the MSSA and MRSA could be separated by Ni‐NTA magnetic nanobeads attached to dCas9 utilizing a magnet. Finally, SYBR Green I (SG I) is added to stain dsDNA and the fluorescence intensity can reflect the concentration of MRSA. It was also confirmed that test samples can be cell lysates without isolation of genomic DNA, this method does not require PCR and therefore is practical and rapid. On the other hand, some researchers have already found a new *mecA* homologue (*mecALGA251*) sharing only 70% nucleotide homology with *mecA,* 34 which may cause false negative results in the test because of specificity of the method. Meanwhile, a large proportion of MRSA strains do not possess *mecA*, and there is a spot of MSSA strains that contains the *mecA* gene^35^, ^36^; this state of affairs make the detection of the *mecA* gene not specific to MRSA.

After the Doudna^37^ lab demonstrated the functions of Cas13a (formerly C2c2), which is responsible for the processing and maturation of crRNA and can degrade non‐targeted RNA after cleavage of targeted RNA directed by RNA. In addition, they found in their research that distinct active sites within the Cas13a protein catalyse pre‐crRNA processing and RNA‐directed RNA cleavage. In addition, they determined that the two distinct catalytic activities of Cas13a can be harnessed together for RNA detection, for which the activated Cas13a is able to cleave thousands of non‐targeted RNAs after cleavage of the target RNA enables potent signal amplification. Feng Zhang also published some papers about the detection ways of nucleic acid called SHERLOCK with attomolar sensitivity and single‐nucleotide mismatch specificity by means of Cas13a in tandem with recombinase polymerase amplification (RPA), which can be coupled with T7 transcription to convert amplified DNA to RNA for subsequent detection.^38^, ^39^, ^40^ The sensitivity of the method combined with RPA is higher than that of any other isothermal amplification method. Cas13a is an RNA‐guided RNase that can cleave the regions complementary with the crRNA thereby providing a platform for specific RNA recognition and cleavage. In addition, after cleaving the target RNA, Cas13a cannot stop cleaving nearby off‐target RNAs. To take advantage of this function, the authors added some reporter RNA which is non‐targeted and will release the signal when it is cut by the Cas13a‐mediated collateral RNA cleavage in the reaction. They uncover that the cleavage products of Cas13a can be activators of Csm6 which is CRISPR type‐III effector nuclease.^41^, ^42^ The synergistic activation of Csm6 by Cas13 can be enhanced by increasing the concentration of activators. Thus, Csm6‐enhanced LwaCas13a amplifies the overall signal in further. This approach can be applied to detecting specific DNA or RNA. It has been proved that this assay can detect specific strains of Zika and Dengue viruses, distinguish pathogenic bacteria, genotype human DNA, and identify cell‐free tumour DNA mutations. With regard to the detection of Zika and Dengue viruses, this method has been tested in patient samples.^40^ They evaluate the performance by comparing SHERLOCK with ZIKV reverse transcription polymerase chain reaction (RT‐PCR) assay in 16 samples from patients and the sensitivity, specificity, concordance are 100%. Also, 24 RT‐PCR–positive DENV RNA samples are confirmed to be positive for DENV using SHERLOCK and the time is less than 2 hours.

The principles of the three methods are quite different and all three involve a combination of the CRISPR/Cas or modified CRISPR/Cas system with some other technique to achieve a detection goal. The main features of the three methods are summarized in Table 1. As for the time and cost, the two methods that involve the CRISPR/Cas9 system take ~3 hours, whereas the dCas9/sgRNA complex combined with FISH is more inexpensive. Regarding sensitivity, 10 colony‐forming units (cfu)/mL is required for detecting MRSA, which is not a low concentration for bacteria. On the other hand, this approach does not require PCR amplification and isolation of genomic DNA; therefore, this method is more convenient than other molecular tests. Meanwhile, sensitivity of the Cas9/sgRNA complex is much higher in the combination with the NASBA amplification technique. It can discriminate between Zika strains because of the distinct PAM sequences, and this assay has superior specificity as compared to non‐PCR‐based methods. With respect to the CRISPR/Cas13a system, it is a promising assay for molecular detection owing to its attomolar sensitivity and single‐base mismatch specificity for recognition of RNA. This assay can detect DNA when T7 transcription is added to transcribe DNA into RNA. Considering that Cas13a cannot stop cleaving the nearby non‐targeted RNAs after recognition and cleavage of the target RNA in bacteria. Recently, researchers tested whether this feature is present in mammalian cells and found that Cas13a cleaves only the target RNA, with the unrelated RNAs intact; this finding may be applied to RNA targeting and manipulations.^43^

**Table 1:** Brief summary of applications in detecting pathogens of three main methods

Main detection system	Combined technique	Time	Cost	Sensitivity	Comparison with other technique	Targeted pathogen	References
Cas9/sgRNA complex	NASBA, sensors for trigger RNA	3 h	$21/test	Discriminate between different Zika strains	Superior specificity compared to non‐PCR‐based methods	Zika determined by the PAM	30
dCas9/sgRNA complex	FISH	2.5 h for cell lysates	Inexpensive (unclear)	10 cfu/mL	Without PCR amplification and isolated genomic DNA	MRSA determined by sgRNA	32
C2c2/Cas13a protein	RPA, T7 transcription	Unclear	$0.61 per test	Attomolar, similar to ddPCR and qPCR	With attomolar sensitivity and single‐base mismatch specificity	Virus, bacteria, genotype human DNA	37, 38

FISH: fluorescence in situ hybridization; NASBA: nuclear acid sequence based amplification; PAM: protospacer adjacent motif; RPA: recombinase polymerase amplification.

## 5 FORESEEABLE BARRIERS TO CRISPR/CAS BASED MOLECULAR DETECTION

Despite the great achievements in CRISPR/Cas technology, there are still many hurdles and limitations in its application of molecular detection. Firstly, the proper recognition of a target site by the sgRNA requires a PAM sequence^2^, ^3^; though this requirement increases the specificity of the system in a way, it has also decreased the flexibility in the selection of target region and the corresponding design of sgRNAs. Furthermore, different kinds of CRISPR types and species have diverse PAM sequence(flanking sequence),^44^ which also complicates the design of sgRNA. Secondly, the “off target” effects which may cause false negative or positive results need to be considered. The frequency of off‐target sgRNA binding varies a lot, ranging from very few off‐targets to great amounts.^25^, ^27^, ^45^, ^46^ However, target sequences can be selected by online software to help reduce the probability of off‐target binding,^47^ and more specific variants of Cas9 protein^48^, ^49^ or CRISPR systems from other types^50^, ^51^ may be helpful to address this issue. Accompanied by the aforementioned off‐targets ameliorated in the future, the accuracy of CRISPR/Cas technology in molecular detection will also be improved. Thirdly, RNA is very fragile due to ubiquitous RNase, the detection of the interested nucleic acid and mutations were prone to be affected. Therefore, it is important to make sure that the designed longer sgRNA for signal amplification of nucleic‐acid detection in CRISPR/Cas9 system is not cut short or degraded by the RNase, which may cause the final result false negative. As for CRISPR/Cas13a detection system, SHERLOCK platform is in good graces for its extremely high sensitivity. Nevertheless, SHERLOCK is an exponential pre‐amplification that saturates quickly after the reaction starts, which makes accurate quantification in real time quite difficult.^39^ More explorations need to be done to observe a proper way about quantification of the detection and to require a wider linearity range. Collectively, further studies to solve these limitations of CRISPR/Cas technology will pave the way for the molecular detection in human diseases in vitro, including different types of cancer.

## 6 CONCLUSIONS

Since the discovery of the CRISPR/Cas system, it has been widely used for genomic editing to treat some mutation‐induced diseases. Nevertheless, there is still too much controversy regarding the applications of CRISPR/Cas9 in medical treatments due to the risk of “off‐target” effects. From Yanfang Fu's^52^ research, we know that off‐target sites that consist of up to five nucleotides difference from the intended target site may be mutagenized by CRISPR/Cas9 at even higher frequencies than the intended on‐target sites. This is a thought‐provoking result meaning that the application of this technique in medicine requires caution because there are countless potential off‐target sites that have four or five mismatches compared with the expected targeted sequence in the human genome. Besides, there are some technical challenges, such as the need for improvement of editing efficacy and selection of delivery methods. Thus, we aim to apply this technique to disease diagnosis because many diseases are caused by a change in gene. We want to combine this technology with some other approaches to detect DNA or RNA, mutations, and SNVs, and the combined method can facilitate the diagnosis of infections with some pathogens and therefore diagnose a disease at the molecular level with greater precision and reliability and without safety concerns.

## CONFLICT OF INTEREST

The authors declare no conflict of interests.
